# Bi_2_Se_3_ Sensitized TiO_2_ Nanotube Films for Photogenerated Cathodic Protection of 304 Stainless Steel Under Visible Light

**DOI:** 10.1186/s11671-018-2717-9

**Published:** 2018-09-21

**Authors:** Wencheng Wang, Xiutong Wang, Ning Wang, Xiaobo Ning, Hong Li, Dongzhu Lu, Xiangju Liu, Qichao Zhang, Yanliang Huang

**Affiliations:** 1 Key Laboratory of Marine Environmental Corrosion and Bio-fouling, Institute of Oceanology, Chinese Academy of Sciences, 7 Nanhai Road, Qingdao, 266071 China; 2 Open Studio for Marine Corrosion and Protection, Pilot National Laboratory for Marine Science and Technology, Qingdao, 266237 China; 3 University of Chinese Academy of Sciences, Beijing, 100049 China; 4 College of Mechanical and Electrical Engineering, Qingdao University, Qingdao, 266071 China; 5 Center for Ocean Mega-Science, Chinese Academy of Sciences, Qingdao, 266071 China

**Keywords:** TiO_2_, Bi_2_Se_3_, Stainless steel, Heterojunction, Photocathodic protection

## Abstract

Titanium dioxide (TiO_2_) nanotube arrays coupled with a narrow gap semiconductor—bismuth selenide (Bi_2_Se_3_)—exhibited remarkable enhancement in the photocathodic protection property for 304 stainless steel under visible light. Bi_2_Se_3_/TiO_2_ nanocomposites were successfully synthesized using a simple two-step method, including an electrochemical anodization method for preparing pure TiO_2_ and a chemical bath deposition method for synthesizing Bi_2_Se_3_ nanoflowers. The morphology and structure of the composite films were studied by scanning electron microscopy, energy dispersion spectroscopy, X-ray photoelectron spectroscopy and X-ray diffraction. In addition, the influence of the Bi_2_Se_3_ content on the photoelectrochemical and photocathodic protection properties of the composite films was also studied. The photocurrent density of the Bi_2_Se_3_/TiO_2_ nanocomposites was significantly higher than that of pure TiO_2_ under visible light. The sensitizer Bi_2_Se_3_ enhanced the efficient separation of the photogenerated electron-hole pairs and the photocathodic protection properties of TiO_2_. Under visible light illumination, Bi_2_Se_3_/TiO_2_ nanocomposites synthesized by the chemical bath deposition method with Bi^3+^ (0.5 mmol/L) exhibited the optimal photogenerated cathodic protection performance for 304 stainless steel.

## Background

As important engineering materials, stainless steels have been widely applied to significant projects in numerous fields due to their excellent corrosion resistance. However, stainless steels can suffer serious corrosion when used in specific aggressive environments, such as acid environments, as well as under chloride-containing or high-temperature conditions [1–4]. Extensive research and applications of the traditional anti-corrosion method, including coatings [5, 6], use of a sacrificial anode [7] and impressed current cathodic protection [8, 9], have been developed during the past few decades. However, eco-friendly and long-lasting anticorrosion technology still remains a major objective. As a new anti-corrosion technology, photocathodic protection was first proposed by Yuan and Tsujikawa in 1995 [10] before receiving attention from corrosion researchers [11–14].

Titanium dioxide (TiO_2_) is an important photoelectric material with good photoelectric conversion and photocatalysis properties and is widely used in catalysts [15], solar cells [16] and gas sensors [17] due to its low cost, non-toxicity and stable chemical properties. TiO_2_ and TiO_2_-based composites are used for photogenerated cathodic protection: a promising technique for corrosion prevention that has undergone rapid development in recent years [18–23]. However, the bandgap (3.2 eV) of TiO_2_ restricts the photoresponse to only the ultraviolet region, which significantly depresses the utilization ratio of solar power. In addition, photo-induced charge carriers in bare TiO_2_ nanoparticles show a very short lifetime due to the rapid recombination of photo-excited electron-hole pairs, which reduces the photocathodic protection effect of pure TiO_2_ films. Thus, how to overcome the above deficiencies of TiO_2_ has become a widely studied topic. Many studies have been conducted on compounding TiO_2_ with non-metal elements (F, N and S) [12, 24, 25], metal atoms (Fe, Co, Cu and Ce) [26–29] and some narrow bandgap nano-semiconductors (Ag_2_O, ZnSe, WO_3_, CdS, Ag_2_S, CdSe and Bi_2_S_3_) [30–36] to improve the carrier separation and light utilization of TiO_2_.

Bismuth selenide (Bi_2_Se_3_) is a direct bandgap layered semiconductor and important member of the V_2_VI_3_ compound family. It has a high absorption coefficient in the visible and near-infrared light regions with a narrow bandgap (0.35 eV) [37]. As an important n-type chalcogenide, Bi_2_Se_3_ possesses many important characteristics, such as a high electrical conductivity [38], appreciable thermoelectric property [39], photosensitivity [40], electrochemical property [41] and photoconductivity [42]. Furthermore, Bi_2_Se_3_ is a popular topological insulator [43–45] and has the unique property of conductive surface states and insulated bulk states. High-quality Bi_2_Se_3_ nanostructures have been prepared using a high vacuum physical deposition method, chemical vapour deposition, atomic layer deposition, pulsed laser deposition and a vapour-liquid-solid technique at high temperature [44–49]. These synthetic methods for Bi_2_Se_3_ require a difficult fabrication, leading to a high production cost. In this paper, the above problems are overcome by employing a low-cost and simple chemical bath deposition method for Bi_2_Se_3_ nanoflower deposition on TiO_2_. The combination of a n-Bi_2_Se_3_/n-TiO_2_ heterojunction as an efficient photoanode was applied to the photogenerated cathodic protection of 304ss for the first time. The morphology, structure and optical absorption property of Bi_2_Se_3_/TiO_2_ nanocomposites were studied by scanning electron microscopy (SEM), X-ray diffraction (XRD), energy-dispersive X-ray spectroscopy (EDS), X-ray photoelectron spectroscopy (XPS) and UV-visible (UV-Vis) diffuse reflectance spectra, respectively.

## Methods

All of the chemicals used in this study were of analytical grade and used as received without further purification. All of the aqueous solutions were prepared using deionized water.

### Preparation of TiO_2_ Film

Ti foils (20 mm × 10 mm × 0.3 mm; > 99.9% purity) were polished using a mixture containing NH_4_F (2.25 g), H_2_O (12.5 mL), H_2_O_2_ (30 wt%, 30 mL) and HNO_3_ (68 wt%, 30 mL), and then, Ti pieces were cleaned with deionized water and ethanol. TiO_2_ film was prepared on Ti foil by the anodic oxidation method reported in the literature [50]. The Pt plate was chosen as the cathode, and the Ti foil was the anode at 20 V for 1 h in an ethylene glycol solution containing NH_4_F (0.22 g), H_2_O (4 mL) and ethylene glycol (40 mL) at ambient temperature. After that, the samples were rinsed with deionized water and ethanol. Finally, the specimens were annealed at 450 °C for 2 h and cooled in ambient air to obtain TiO_2_ film.

### Synthesis of Bi_2_Se_3_ on the TiO_2_ Film

The Bi_2_Se_3_ was prepared by the chemical bath deposition method. In the experimental procedure, 8 mmol of nitrilotriacetic acid (H_3_NTA) and 0.4 mmol of Bi(NO_3_)_3_·5H_2_O were added to deionized water (400 mL) to form the bismuth chelate, with a Bi^3+^ concentration of 1.0 mmol/L in the mixed solution. Two millimoles of ascorbic acid as the reducing reagent was added to the above solution, and then, ammonium hydroxide was cautiously added, dropwise, until the pH of the mixture was adjusted to approximately 8.6~8.9 and mixed solution appeared colourless and transparent. Finally, Na_2_SeSO_3_ (20 mL, 30 mmol/L) was injected into the above solution. In all of the above experiments, the aqueous solutions were thoroughly stirred with a magnetic stirrer to obtain a homogeneous solution. Then, a TiO_2_ substrate was immersed in the final solution (40 mL) in a beaker (100 mL). The beaker covered with cling film was then transferred into an oven heated to a temperature of 80 °C for 200 min to obtain the Bi_2_Se_3_ nanoflower on the TiO_2_ substrate. Finally, the sample was removed from the beaker and washed several times with deionized water and ethanol and then allowed to dry in ambient air. In this way, Bi_2_Se_3_-sensitized TiO_2_ films were obtained and labelled with Bi_2_Se_3_/TiO_2_-1.0. For simplicity, different quantities of Bi_2_Se_3_ on TiO_2_ substrates are designated as Bi_2_Se_3_/TiO_2_-γ in this paper, where γ denotes the concentration of Bi^3+^ in the H_3_NTA and Bi (NO_3_)_3_·5H_2_O solution. With the quantities of the other reagents held constant, Bi_2_Se_3_/TiO_2_-0.5 and Bi_2_Se_3_/TiO_2_-0.25 were obtained for Bi^3+^concentrations of 0.5 mmol/L and 0.25 mmol/L, respectively. The influence of different quantities of Bi_2_Se_3_ on the photoelectrochemical and photocathodic protection properties of the composite films was investigated in this paper.

### Morphology and Composition Analysis

Scanning electron microscopy (SEM, Hitachi S-4800, Japan) was used to investigate the morphologies of the prepared films. Energy-dispersive X-ray spectroscopy (EDS, Oxford Energy 350 X-ray energy spectrum analyser) and X-ray photoelectron spectroscopy (XPS, Thermo Scientific ESCALAB 250Xi) were employed to determine the chemical composition of the Bi_2_Se_3_/TiO_2_ nanocomposites. UV-Vis DRS (Japan Hitachi UH4150) was used to determine the light absorbance of the samples. The crystalline phase composition of the samples was characterised by an X-ray diffractometer (XRD, Germany Bruker AXSD8) using Cu K_α_ radiation (γ = 1.54056 Å) from 10° to 80°.

### Photoelectrochemical Measurements

**Fig. 1:**
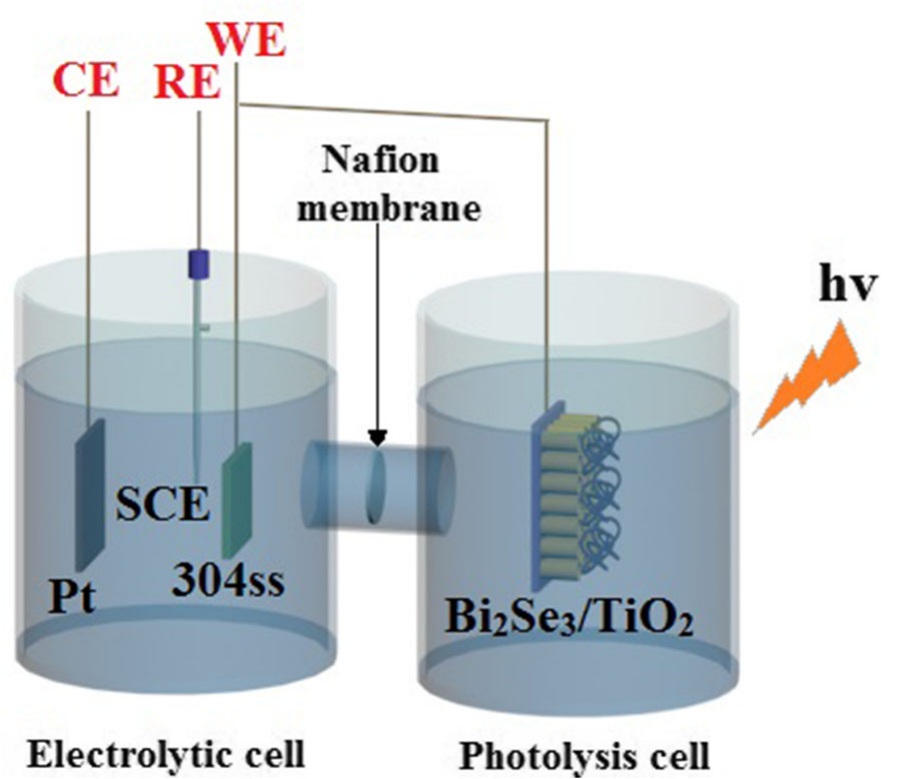
Schematic sketches of the experimental setup used for photoelectrochemical analysis

As shown in Fig. 1, a coupling system comprising photolysis and electrolytic cells was used for the photoelectrochemical measurements, and a proton exchange membrane was used to link the two cells together. The photolysis cell contained a 0.1 mol/L Na_2_S and 0.2 mol/L NaOH mixed solution, which played the role of a sacrificial agent for promoting the separation of electrons and holes [33, 51], while a 0.5 mol/L NaCl solution was used as the electrolyte for the electrolytic cell. In the electrolytic cell, a three-electrode system was adopted with a Pt foil as the counter electrode (CE), saturated calomel electrode as the reference electrode (RE) and 304ss as the working electrode (WE). Bi_2_Se_3_/TiO_2_ nanocomposite samples placed in the photolysis cell were connected to a 304ss electrode immersed in the electrolytic cell by a copper wire. The light source in the visible light range was irradiated by a high-pressure xenon lamp (PLS-SXE 300 C, Beijing Perfectlight Company, China). The changes in the open-circuit potential (OCP) and photocurrent curves were measured using a Gamry potentiostat/galvanostat/ZRA system (GAMRY 3000, Gamry Instruments, USA) before and during light irradiation.

## Results and Discussion

### Characterization of Pure TiO2 and Bi2Se3/TiO2

**Fig. 2:**
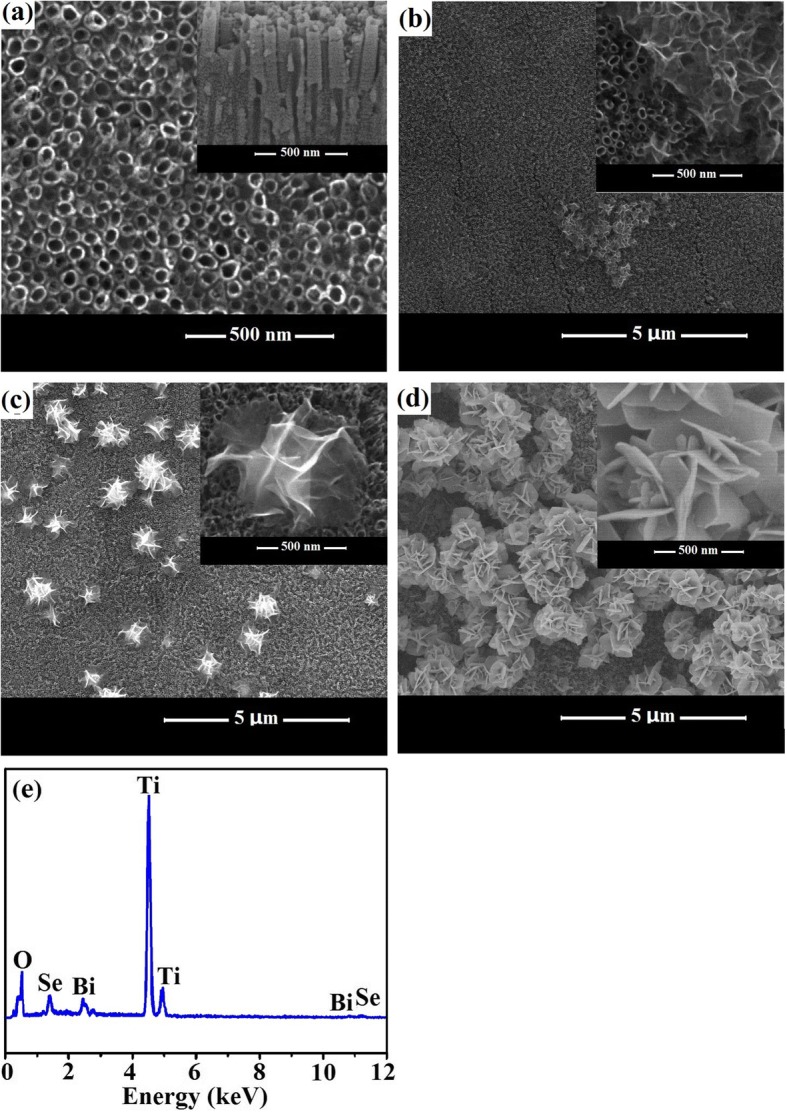
SEM images for **a** pure TiO_2_, **b** Bi_2_Se_3_/TiO_2_-0.25, **c** Bi_2_Se_3_/TiO_2_-0.5 and **d** Bi_2_Se_3_/TiO_2_-1.0; **e** EDS spectrum for Bi_2_Se_3_/TiO_2_-0.5 films

Figure 2a shows typical top view and cross-sectional topographies for TiO_2_ films prepared under the anodization method. The TiO_2_ nanotube arrays show a nanoporous structure composed of well-ordered and high-density nanotubes with an average inner diameter and length of approximately 55 nm and 680 nm, respectively. As shown in Fig. 2b–d, the TiO_2_ nanotube surfaces were successfully modified by Bi_2_Se_3_ via the chemical bath deposition method for different concentrations of Bi^3+^. For Bi_2_Se_3_/TiO_2_-0.25, the Bi_2_Se_3_ nanoflakes were sporadically distributed and aggregated unevenly across the TiO_2_ nanotubes (Fig. 2b). When the concentration of Bi^3+^ was 0.5 mmol/L, Bi_2_Se_3_ was composed almost entirely of flower-like patterns of pliable ultrathin nanoflakes with a diameter of approximately 800 nm, without blocking the nozzle of the TiO_2_ nanotubes or damaging them (Fig. 2c). Bi_2_Se_3_ nanoflowers that were observed to be evenly distributed on the surface of the TiO_2_ showed an internal cross-linked structure for the ultrathin nanoflakes, which effectively prevented lamella aggregation and maintained a long-standing lifetime of the architectures, as shown in Fig. 2c. After the concentration of Bi^3+^ was increased to 1.0 mmol/L, the amount and diameter of the Bi_2_Se_3_ nanoflowers significantly increased, and the agglomeration of nanoflowers blocked the nanotubes, as shown in Fig. 2d. The corresponding EDS spectrum of the Bi_2_Se_3_/TiO_2_-0.5 films shown in Fig. 2e revealed that the characteristic peaks for Ti, O, Bi and Se were marked with atomic percentages of Bi and Se of 0.9% and 1.3%, respectively. It is well known that the measurement error of EDS test is increased with the decrease of content of test element. So, it is acceptable that the atomic ratio of Bi and Se is close to 2:3.

**Fig. 3:**
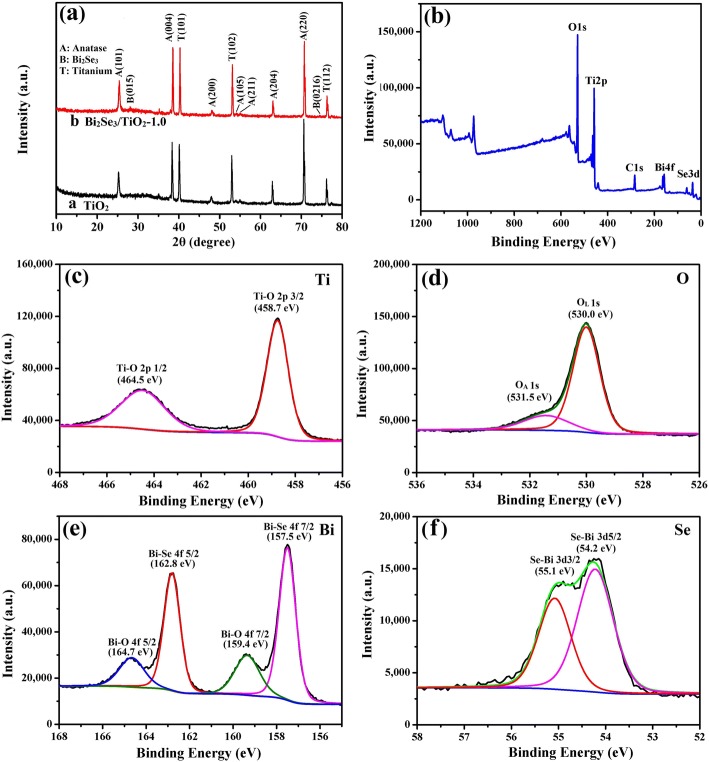
**a** XRD patterns for pure TiO_2_ and Bi_2_Se_3_/TiO_2_-1.0 nanocomposites; **b** the total survey spectrum, **c** Ti 2p, **d** O 1s, **e** Bi 4f and **f** Se 3d XPS spectra for Bi_2_Se_3_/TiO_2_-1.0 nanocomposites

Figure 3a shows the XRD spectra for pure TiO_2_ (curve a) and Bi_2_Se_3_/TiO_2_-1.0 nanocomposites (curve b). Aside from the Ti substrate peaks, the diffraction peaks at 25.38°, 38.03°, 48.01°, 54.05°, 55.17°, 62.71° and 70.44° were well matched with the lattice planes (101), (004), (200), (105), (211), (204) and (220) of anatase TiO_2_, respectively (JCPDS 21-1272). Except for the TiO_2_ peaks, the distinctive diffraction peaks at 29.35° and 74.90° were indexed to the lattice planes (015) and (0216) of the rhombohedral crystal Bi_2_Se_3_ (JCPDS 33-0214). However, there is no obvious peak of Bi_2_Se_3_/TiO_2_-1.0 due to low content Bi_2_Se_3_ deposited on TiO_2_ and the XRD spectra conformed to the SEM and EDS results. X-ray photoelectron spectroscopy (XPS) was used to further determine the chemical compositions and states of the Bi_2_Se_3_/TiO_2_ nanocomposites. As shown in Fig. 3b, XPS revealed the existence of Bi, Se, Ti and O components in addition to C contaminants due to adventitious hydrocarbon contamination. Figure 3c shows the peak positions for Ti 2p at 458.7 and 464.5 eV, indicating that the titanium oxides mainly consisted of TiO_2_ [52]. As illustrated in Fig. 3d, the O 1s semaphores matched with two Gaussian peaks: the maximum at the lower binding energy (530.0 eV) was attributed to the lattice oxygen (O_L_) in Bi_2_Se_3_/TiO_2_ nanocomposites and the second at the higher binding energy (531.5 eV) was derived from the adsorbed oxygen (O_A_), including weak bonding oxygen or hydroxyl groups. The existence of O_A_ was due to the generation of oxygen vacancies on the surface of the nanocomposites, which might improve the photoelectric conversion properties of Bi_2_Se_3_/TiO_2_ nanocomposites in photocathodic protection [53]. Figure 3e shows that the 4f_7/2_ asymmetric peak for Bi resolved into two peaks (157.5 and 159.4 eV), with the Bi 4f_5/2_ spectrum similarly divided into two bands at 162.8 and 164.7 eV, respectively. The positions of the lower peaks (157.5 eV and 162.8 eV) were in good agreement with those in Bi_2_Se_3_, with the higher peaks corresponding to bismuth oxide at 159.4 eV and 164.7 eV [54, 55]. It can be concluded that a handful of bismuth metal was oxidized during the synthetic process with Bi_2_Se_3_ modifying pure TiO_2_. As shown in Fig. 3f, the two peaks were assigned to the 3d_3/2_ and 3d_5/2_ core levels of Se at 55.1 and 54.2 eV, respectively, indicating that Se existed in the form of Se^2-^ [56].

**Fig. 4:**
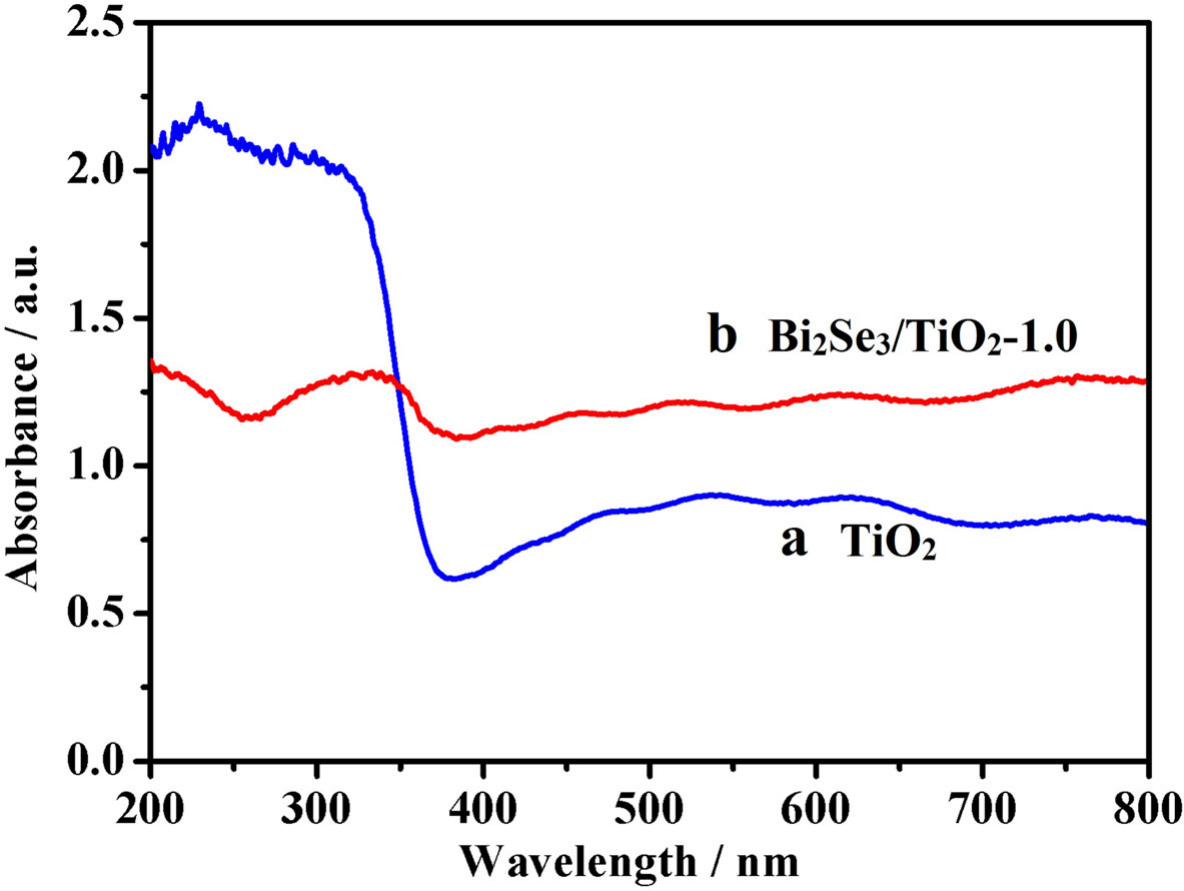
UV-visible absorption spectra for TiO_2_ (a) and Bi_2_Se_3_/TiO_2_-1.0 (b)

Figure 4 shows the light absorption abilities of pure TiO_2_ and Bi_2_Se_3_/TiO_2_-1.0 nanocomposites. The characteristic absorption edge for pure TiO_2_ was approximately 380 nm within the UV region due to the bandgap energy of anatase TiO_2_ (3.2 eV) (curve a). Pronounced adsorption was observed for Bi_2_Se_3_/TiO_2_ nanocomposites in the visible light region (350–800 nm) (curve b), with visible light absorption abilities higher than those of pure TiO_2_ due to the incorporation of the Bi_2_Se_3_ nanoflower. This phenomenon can be ascribed to the fact that Bi_2_Se_3_ is excited under visible light due to its narrow bandgap (0.35 eV), with electrons and holes produced in its conduction band (CB) and valence band (VB). Therefore, the addition of Bi_2_Se_3_ effectively increases the visible light absorption capability of Bi_2_Se_3_/TiO_2_ nanocomposites.

### Photocathodic Protection Performance of Pure TiO2 and Bi2Se3/TiO2

**Fig. 5:**
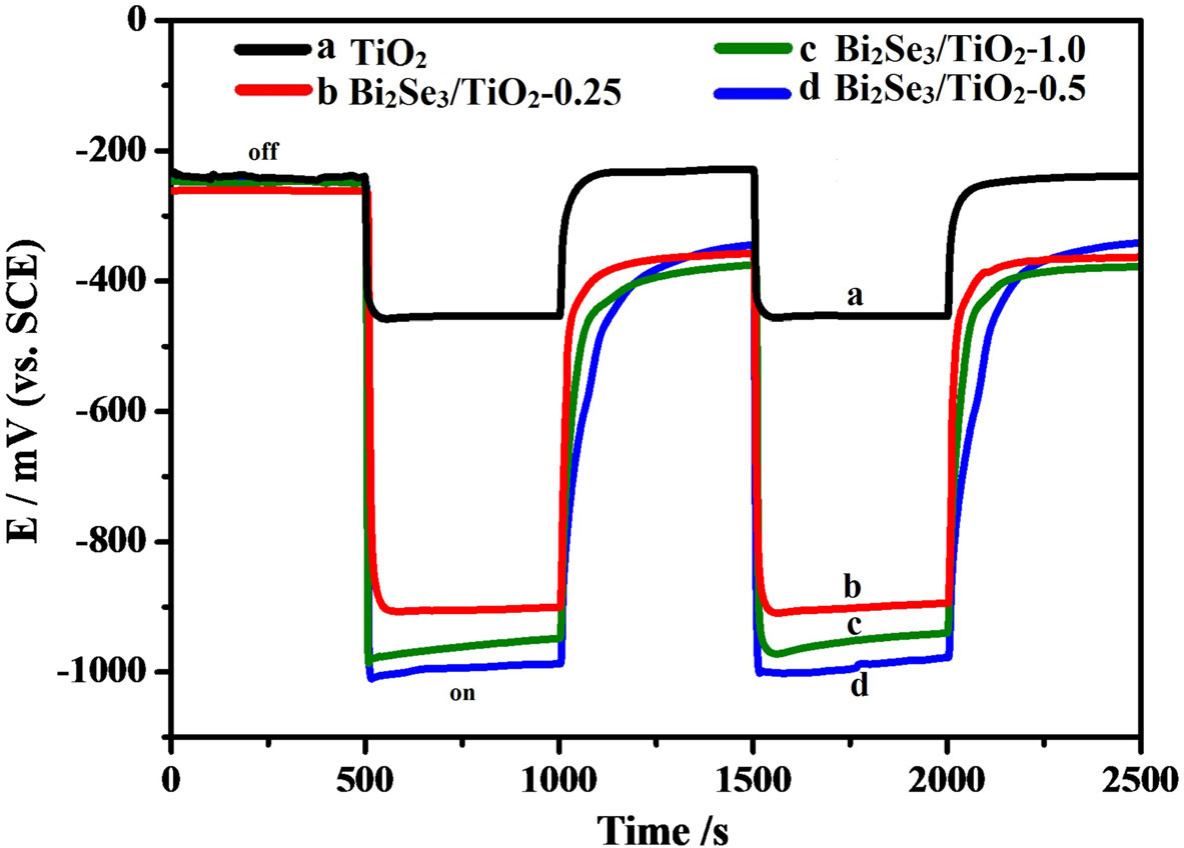
OCP for 304ss coupled with pure TiO_2_ and Bi_2_Se_3_/TiO_2_ nanocomposites in a 0.5 mol NaCl solution

As shown in Fig. 5, the OCP curves for 304ss coupled with pure TiO_2_ and Bi_2_Se_3_/TiO_2_ nanocomposite photoanodes were measured under intermittent visible light, with the OCP response to illumination prompted and shifted to a negative potential for all of the coupled electrodes. At the initial phase of light on, the OCP for all of the coupled electrodes showed a negative shift over a short time, which was due to the transfer of the excited photoelectrons from the pure TiO_2_ and Bi_2_Se_3_/TiO_2_ nanocomposite to the 304ss electrode [1, 57]. Subsequently, the relatively stable OCP values can be attributed to the balancing rate between the creation and recombination of photogenerated electrons [32]. After switching off the irradiation, the OCP values for the Bi_2_Se_3_/TiO_2_ nanocomposites returned to their original values at a slower speed compared to pure TiO_2_. This phenomenon might be attributed to the electron pool effect of Bi_2_Se_3_/TiO_2_ nanocomposites, which can store photoinduced electrons under light irradiation and slowly release these electrons without light irradiation. Under visible light irradiation, the OCP value for 304ss was approximately − 450 mV when coupled with TiO_2_ (curve a), and the OCP values for 304ss coupled with Bi_2_Se_3_/TiO_2_-0.25 (curve b), Bi_2_Se_3_/TiO_2_-0.5 (curve d) and Bi_2_Se_3_/TiO_2_-1.0 (curve c) reached − 905 mV, − 996 mV and − 958 mV, respectively. These results indicated that 304ss was cathodically polarized once coupled with Bi_2_Se_3_/TiO_2_ nanocomposites and that a good cathodic protection for 304ss might be provided by the Bi_2_Se_3_/TiO_2_ photoanodes. As shown in Fig. 5 d, the 304ss coupled to Bi_2_Se_3_/TiO_2_-0.5 possessed most negative potential indicated that the best photocathodic protection performance for 304ss. This result might be because the active sites and light harvesting increased with the increasing Bi_2_Se_3_ content. However, an excessive amount of Bi_2_Se_3_ particles served as the recombination sites for electrons and holes, which hindered the charge transfer from the Bi_2_Se_3_/TiO_2_ nanocomposites to 304ss.

**Fig. 6:**
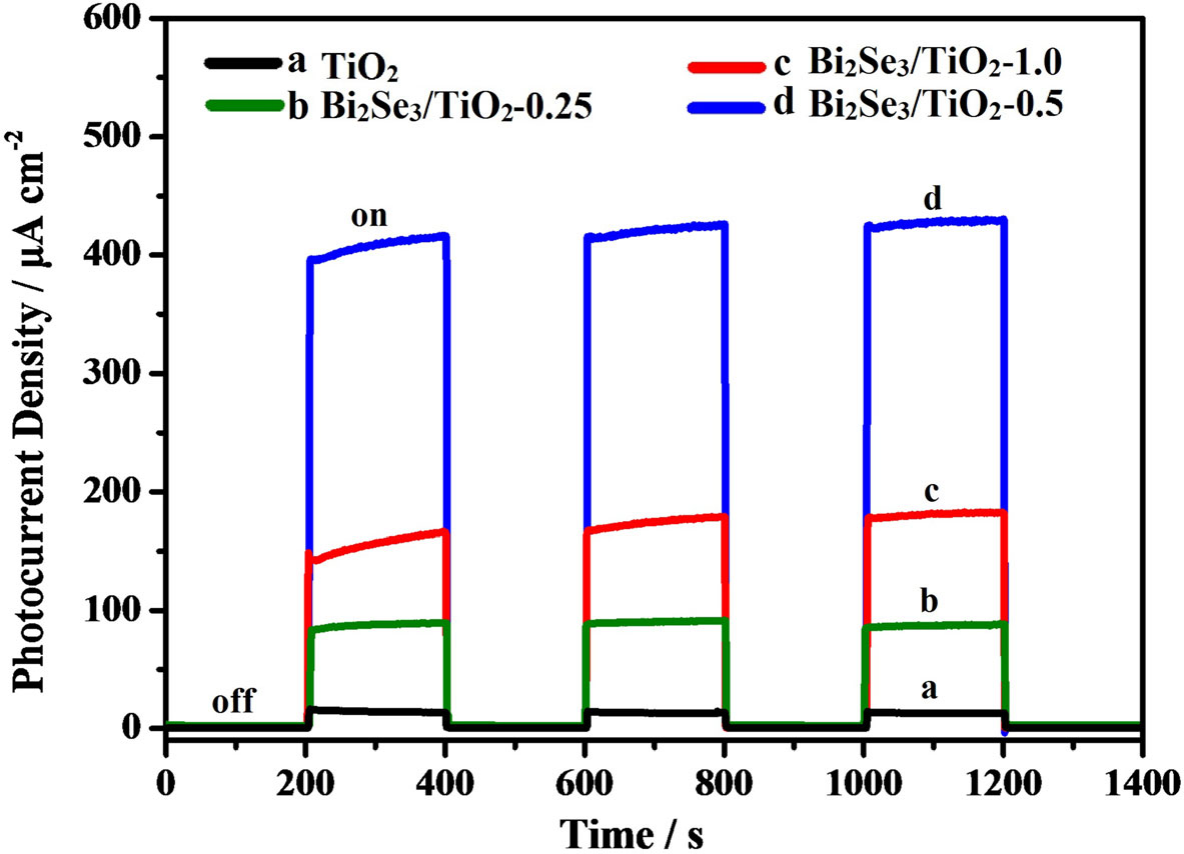
Photocurrent density vs. time curves for pure TiO_2_ and Bi_2_Se_3_/TiO_2_ nanocomposites in 0.1 mol/L Na_2_S and 0.2 mol/L NaOH mixed solution

As shown in Fig. 6, the photocurrent density vs. time curves for TiO_2_ and Bi_2_Se_3_/TiO_2_ nanocomposites showed a rapid and reproducible photoresponse under intermittent visible light illumination, which reflected the photoelectric conversion performance of the materials. The photogenerated current was relatively small under visible light due to weak visible light absorption (curve a). However, the photogenerated current increased remarkably under visible light illumination following sensitization of TiO_2_ by the Bi_2_Se_3_ nanoflower (curves b to d). The data implied that the Bi_2_Se_3_/TiO_2_ nanocomposites were capable of utilizing visible light and that the heterojunction between TiO_2_ and Bi_2_Se_3_ promoted the separation of photogenerated electrons and holes [58]. Furthermore, the photoelectrons produced in the conduction band of the Bi_2_Se_3_ nanoflower can be easily transferred to the more positive conduction band of the TiO_2_ nanotubes under visible light illumination. After three irradiation interval, the photocurrent maintained a relatively steady value and no photocurrent degradation was detected, illustrating the good photochemical stability of the Bi_2_Se_3_/TiO_2_ nanocomposite films. For different concentrations of Bi^3+^, the Bi_2_Se_3_/TiO_2_ nanocomposites showed different intensities for the photocurrent response. In particular, the transient photocurrent density for Bi_2_Se_3_/TiO_2_-0.5 (415 μA/cm^2^) was higher than that for Bi_2_Se_3_/TiO_2_-0.25 (85 μA/cm^2^) and Bi_2_Se_3_/TiO_2_-1.0 (160 μA/cm^2^), indicating that Bi_2_Se_3_/TiO_2_-0.5 possessed an ideal separation efficiency for the photogenerated electron-hole pairs. The active sites and light harvesting were decreased because of the deficiency of Bi_2_Se_3_ nanoflowers on the Bi_2_Se_3_/TiO_2_ nanocomposite films, while recombination sites for electrons and holes increased in the presence of an excessive amount of Bi_2_Se_3_ nanoflowers. Under visible light illumination, the largest photoinduced current density of the Bi_2_Se_3_/TiO_2_-0.5 photoanode was consistent with the largest photoinduced potential drops illustrated in Fig. 5, further validating the optimal photocathodic protection performance of Bi_2_Se_3_/TiO_2_-0.5 for 304ss.

**Fig. 7:**
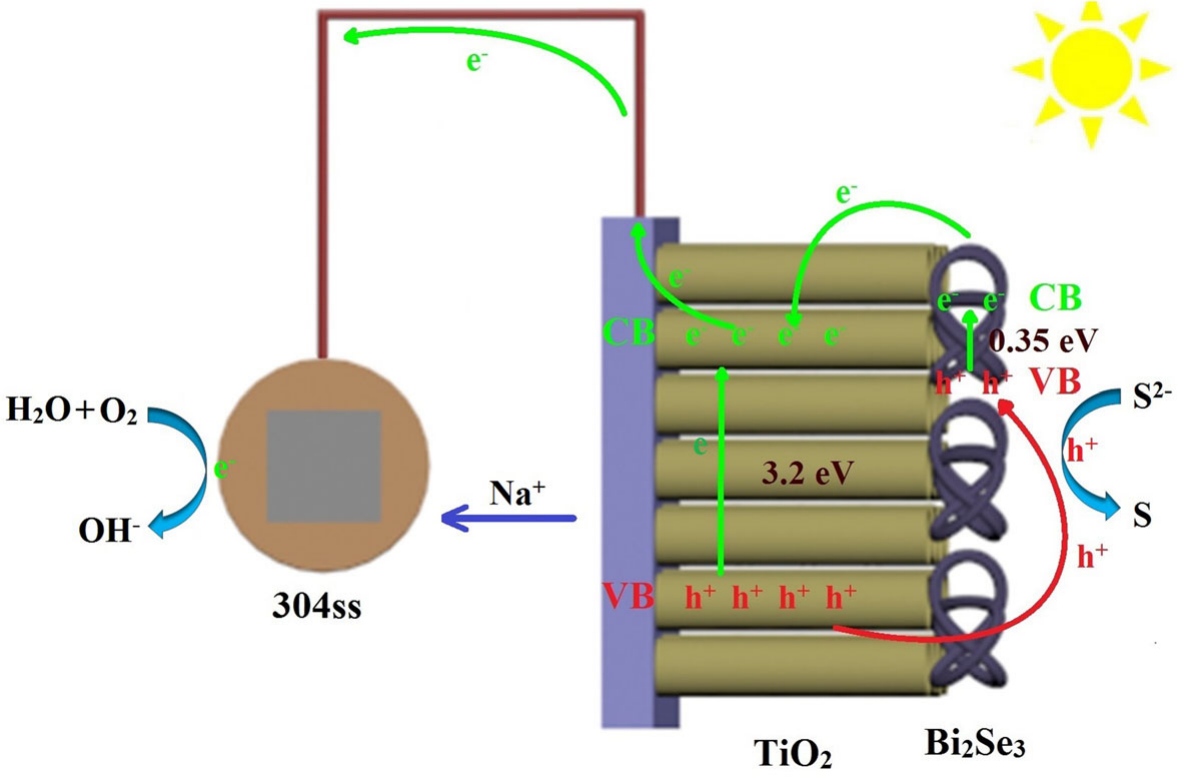
Schematic representation of the electron transfer processes in Bi_2_Se_3_/TiO_2_

Figure 7 shows the photoelectric conversion and transportation processes for the Bi_2_Se_3_/TiO_2_ nanocomposites. Under visible light, Bi_2_Se_3_ nanoflowers can readily absorb photons as they contain adsorbed oxygen (O_A_) and have a narrow bandgap width (0.35 eV). When the photons are absorbed by the Bi_2_Se_3_ nanoflowers, photoexcited electrons will be generated by excitation from the valence band (VB) of Bi_2_Se_3_ to the conduction band (CB) of Bi_2_Se_3_. The photoexcited electrons in the CB of Bi_2_Se_3_ are shifted to the CB of TiO_2_, while the photogenerated holes in the VB of TiO_2_ are transferred to the VB of Bi_2_Se_3_, and then are captured by S^2−^ in the electrolyte to turn into S on the surface of photoanode film. When the photoexcited electrons exit the photoanode and transfer to 304ss, they will react with the oxygen gas and water to convert OH^−^. Furthermore, Na^+^ is transported from electrolytic cell to photolysis cell by proton exchange membrane, so that the coupling system is electrically neutral as a whole. As a consequence, the photogenerated charges are effectively separated and the recombination probability for photogenerated electron-hole pairs is reduced. Once 304ss receives photoexcited electrons from the Bi_2_Se_3_/TiO_2_ nanocomposite through the wire, the potential of 304ss shifts negatively. Under visible light illumination, the Bi_2_Se_3_/TiO_2_ nanocomposites can reduce the corrosion rate of 304ss. Therefore, the efficient separation of photo-excited electron-hole pairs in Bi_2_Se_3_/TiO_2_ nanocomposites will accelerate the redox reaction and generate effective photocathodic protection for 304ss.

## Conclusions

In this paper, TiO_2_ nanotube arrays were prepared by the anodization method and Bi_2_Se_3_ nanoflowers were grown on TiO_2_ nanotubes by chemical bath deposition. The Bi_2_Se_3_/TiO_2_ nanocomposites showed a homogeneous distribution and ordered characteristics. Electrochemical tests for the nanocomposites and pure TiO_2_ coupled with 304ss showed that the photogenerated cathodic protection performance of the Bi_2_Se_3_/TiO_2_ nanocomposites was superior compared to that for pure TiO_2_. The OCP value for 304ss coupled with Bi_2_Se_3_/TiO_2_-0.5 showed a negative shift to − 996 mV under visible light illumination due to the active sites and light harvesting of TiO_2_ sensitized by Bi_2_Se_3_. By comparing the results of the electrochemical tests for three Bi_2_Se_3_/TiO_2_ nanocomposites, the nanocomposite prepared using 0.5 mmol/L Bi^3+^ in the electrolyte exhibited optimal performance.

## Abbreviations

304ss: 304 stainless steel
CB: Conduction band
CE: The counter electrode
EDS: Energy-dispersive X-ray spectroscopy
H_3_NTA: Nitrilotriacetic acid
OA: Adsorbed oxygen
OCP: Open-circuit potential
OL: Lattice oxygen
RE: The reference electrode
SCE: Saturated calomel electrode
SEM: Scanning electron microscopy
UV-Vis: UV-visible diffuse reflectance spectra
VB: Valence band
WE: The working electrode
XPS: X-ray photoelectron spectroscopy
XRD: X-ray diffraction
